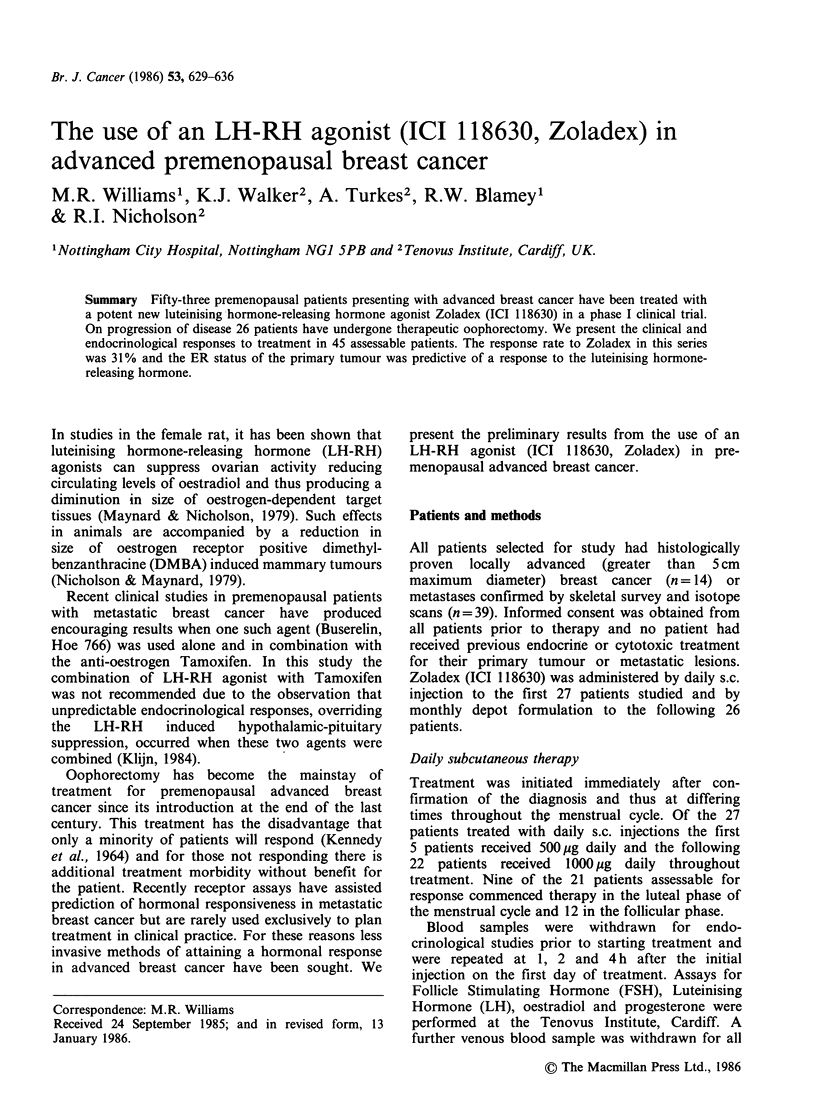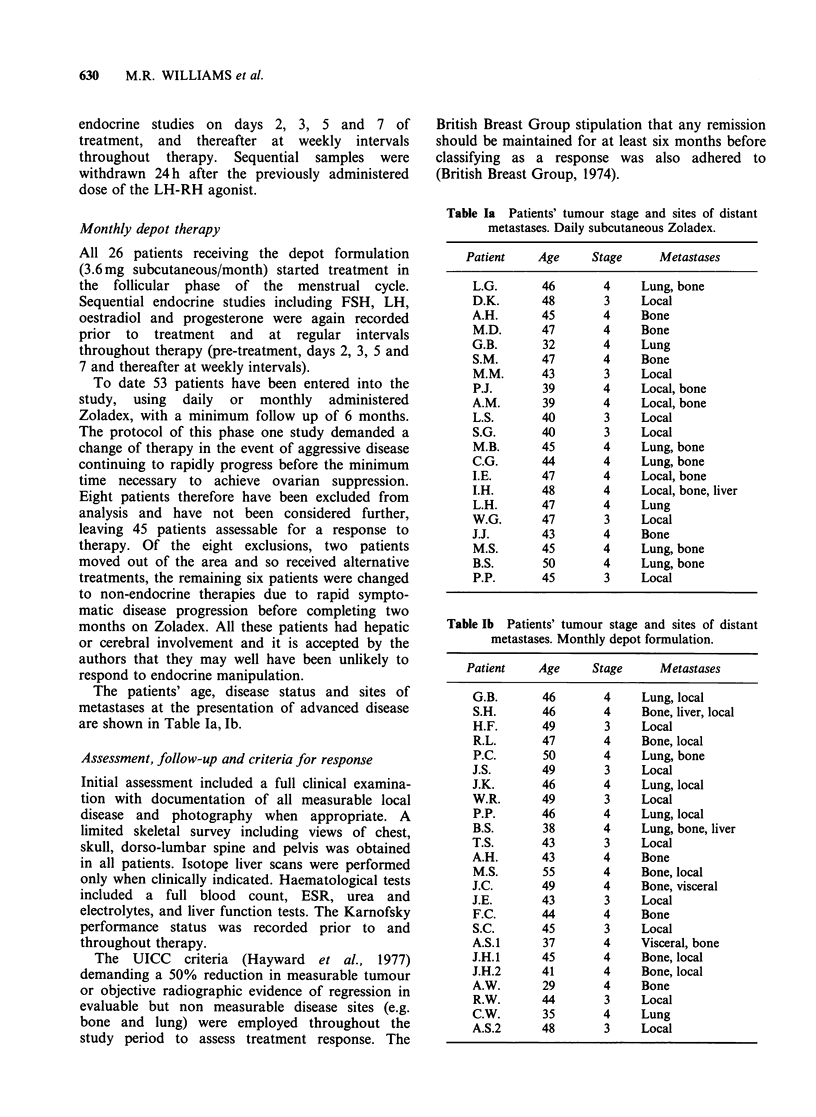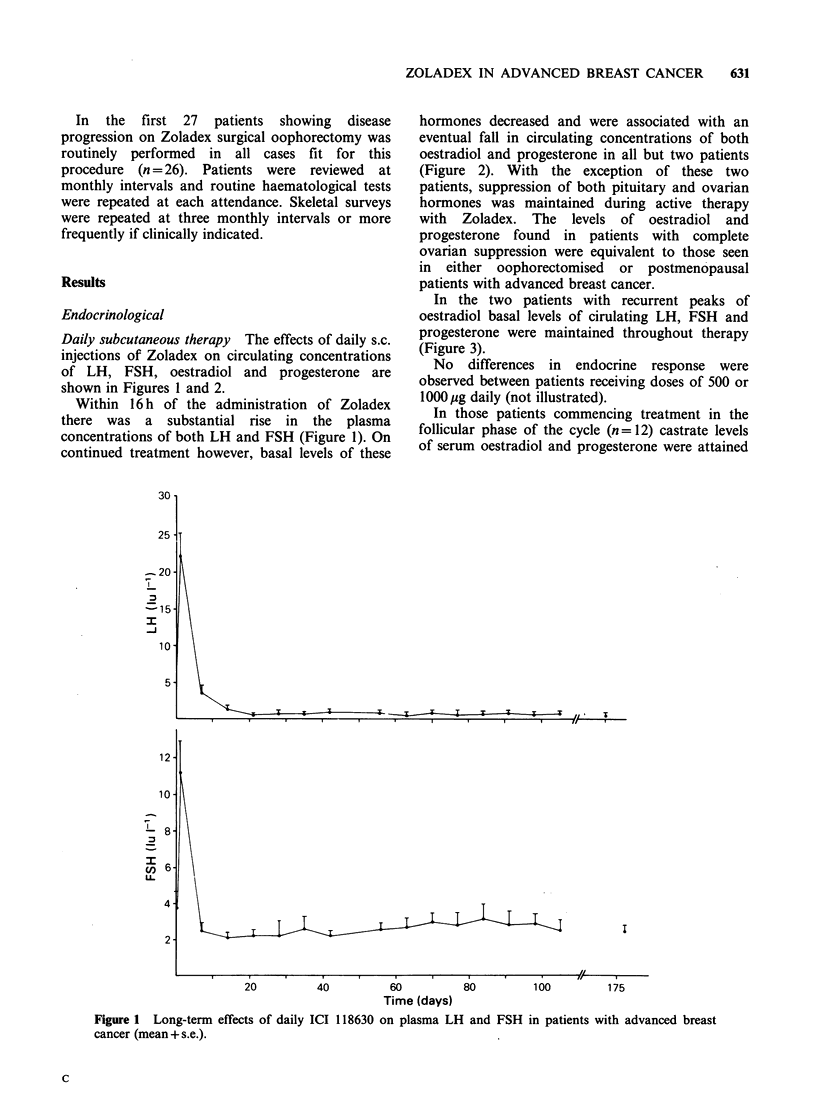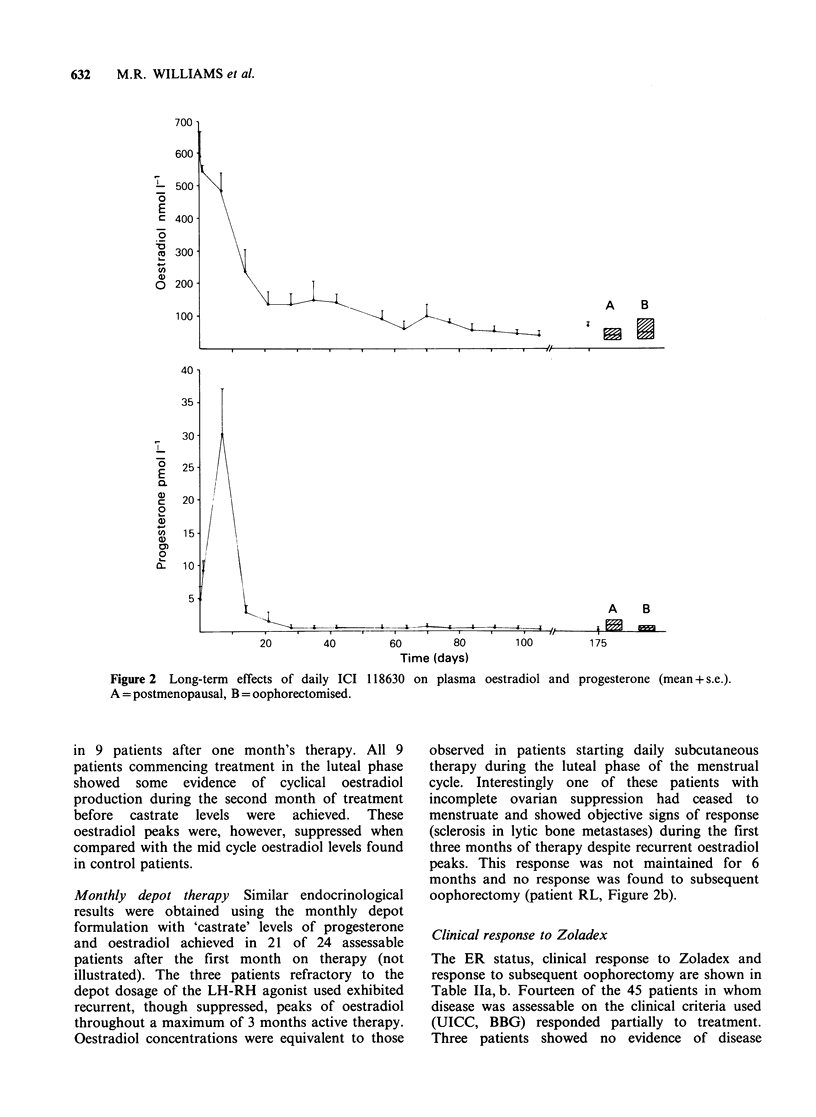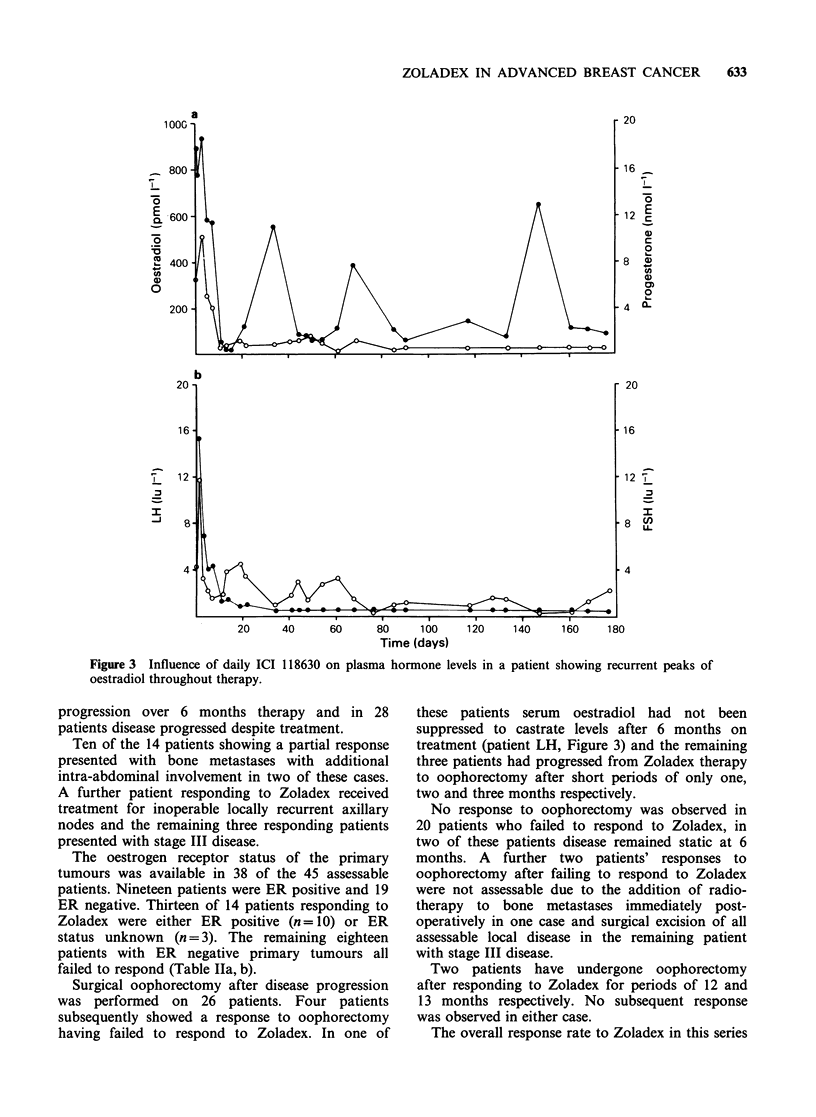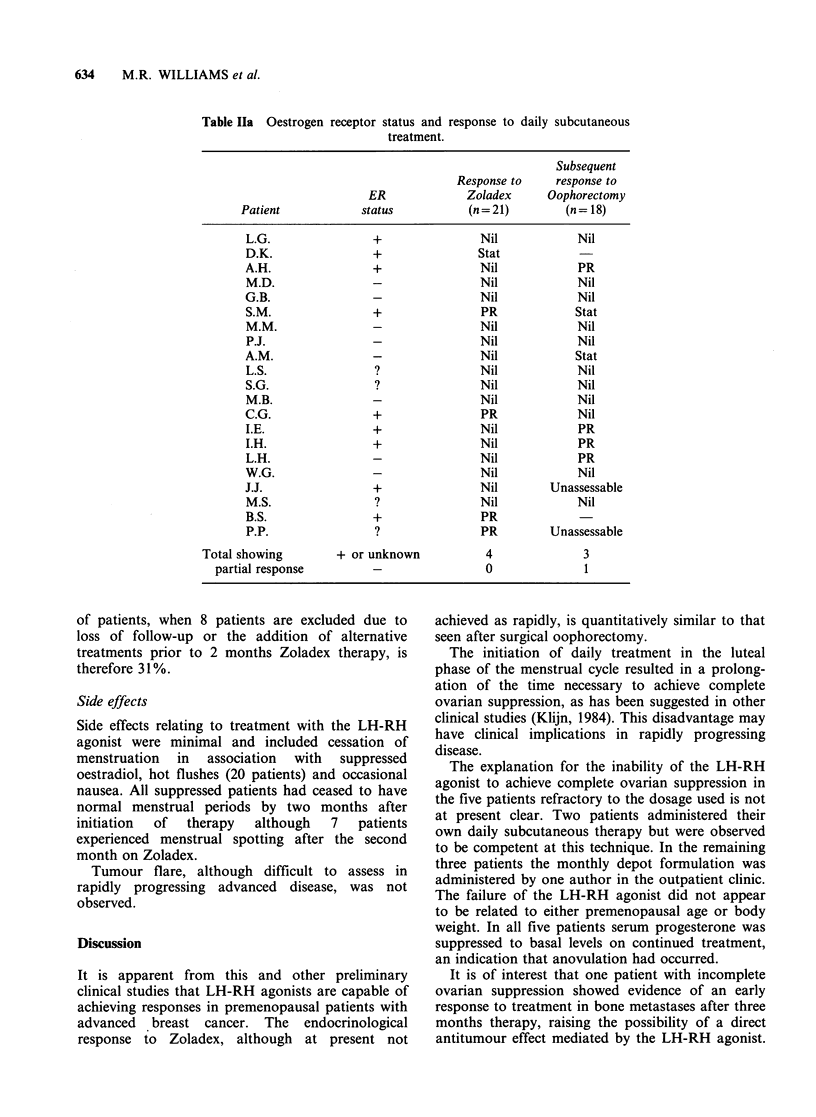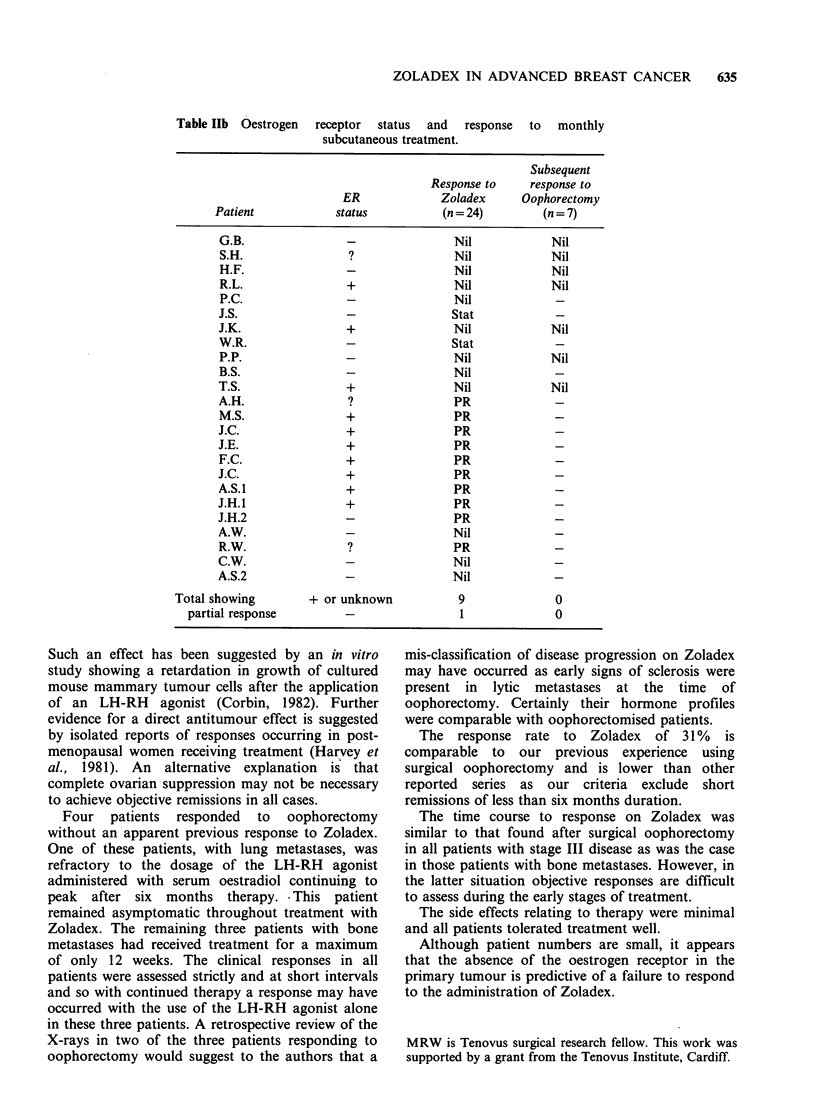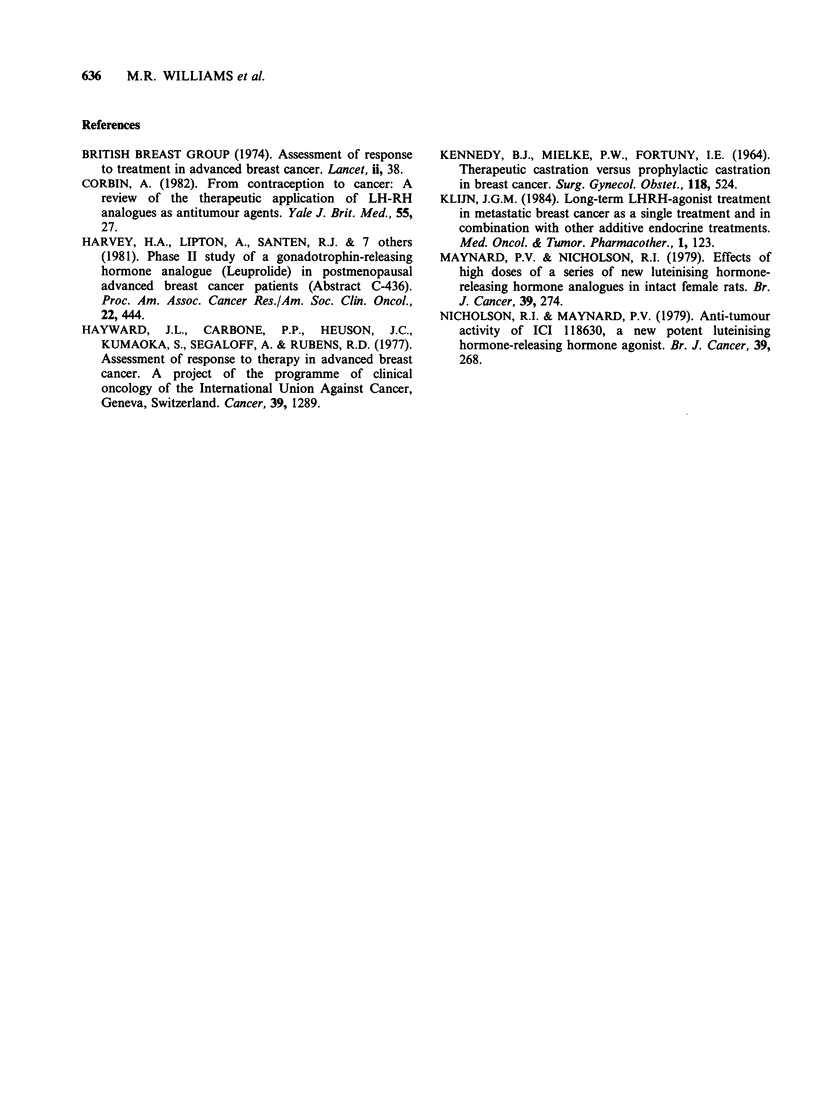# The use of an LH-RH agonist (ICI 118630, Zoladex) in advanced premenopausal breast cancer.

**DOI:** 10.1038/bjc.1986.106

**Published:** 1986-05

**Authors:** M. R. Williams, K. J. Walker, A. Turkes, R. W. Blamey, R. I. Nicholson

## Abstract

Fifty-three premenopausal patients presenting with advanced breast cancer have been treated with a potent new luteinising hormone-releasing hormone agonist Zoladex (ICI 118630) in a phase I clinical trial. On progression of disease 26 patients have undergone therapeutic oophorectomy. We present the clinical and endocrinological responses to treatment in 45 assessable patients. The response rate to Zoladex in this series was 31% and the ER status of the primary tumour was predictive of a response to the luteinising hormone-releasing hormone.


*Br. J. Cancer* (1986) **53**, 629–636

### The use of an LH-RH agonist (ICI 118630, Zoladex) in advanced premenopausal breast cancer

M.R. Williams[1], K.J. Walker[2], A. Turkes[2], R.W. Blamey[1] & R.I. Nicholson[2]

[1]*Nottingham City Hospital, Nottingham NG1 5PB and* [2]*Tenovus Institute, Cardiff, UK.*

**Summary** Fifty-three premenopausal patients presenting with advanced breast cancer have been treated with a potent new luteinising hormone-releasing hormone agonist Zoladex (ICI 118630) in a phase I clinical trial. On progression of disease 26 patients have undergone therapeutic oophorectomy. We present the clinical and endocrinological responses to treatment in 45 assessable patients. The response rate to Zoladex in this series was 31% and the ER status of the primary tumour was predictive of a response to the luteinising hormone-releasing hormone.

In studies in the female rat, it has been shown that luteinising hormone-releasing hormone (LH-RH) agonists can suppress ovarian activity reducing circulating levels of oestradiol and thus producing a diminution in size of oestrogen-dependent target tissues (Maynard & Nicholson, 1979). Such effects in animals are accompanied by a reduction in size of oestrogen receptor positive dimethylbenzanthracine (DMBA) induced mammary tumours (Nicholson & Maynard, 1979).

Recent clinical studies in premenopausal patients with metastatic breast cancer have produced encouraging results when one such agent (Buserelin, Hoe 766) was used alone and in combination with the anti-oestrogen Tamoxifen. In this study the combination of LH-RH agonist with Tamoxifen was not recommended due to the observation that unpredictable endocrinological responses, overriding the LH-RH induced hypothalamic-pituitary suppression, occurred when these two agents were combined (Klijn, 1984).

Oophorectomy has become the mainstay of treatment for premenopausal advanced breast cancer since its introduction at the end of the last century. This treatment has the disadvantage that only a minority of patients will respond (Kennedy *et al.*, 1964) and for those not responding there is additional treatment morbidity without benefit for the patient. Recently receptor assays have assisted prediction of hormonal responsiveness in metastatic breast cancer but are rarely used exclusively to plan treatment in clinical practice. For these reasons less invasive methods of attaining a hormonal response in advanced breast cancer have been sought. We present the preliminary results from the use of an LH-RH agonist (ICI 118630, Zoladex) in premenopausal advanced breast cancer.

Correspondence: M.R. Williams
Received 24 September 1985; and in revised form, 13 January 1986.

### Patients and methods

All patients selected for study had histologically proven locally advanced (greater than 5 cm maximum diameter) breast cancer ($n=14$) or metastases confirmed by skeletal survey and isotope scans ($n=39$). Informed consent was obtained from all patients prior to therapy and no patient had received previous endocrine or cytotoxic treatment for their primary tumour or metastatic lesions. Zoladex (ICI 118630) was administered by daily s.c. injection to the first 27 patients studied and by monthly depot formulation to the following 26 patients.

### *Daily subcutaneous therapy*

Treatment was initiated immediately after confirmation of the diagnosis and thus at differing times throughout the menstrual cycle. Of the 27 patients treated with daily s.c. injections the first 5 patients received 500 $\mu$g daily and the following 22 patients received 1000 $\mu$g daily throughout treatment. Nine of the 21 patients assessable for response commenced therapy in the luteal phase of the menstrual cycle and 12 in the follicular phase.

Blood samples were withdrawn for endocrinological studies prior to starting treatment and were repeated at 1, 2 and 4 h after the initial injection on the first day of treatment. Assays for Follicle Stimulating Hormone (FSH), Luteinising Hormone (LH), oestradiol and progesterone were performed at the Tenovus Institute, Cardiff. A further venous blood sample was withdrawn for all

© The Macmillan Press Ltd., 1986

endocrine studies on days 2, 3, 5 and 7 of treatment, and thereafter at weekly intervals throughout therapy. Sequential samples were withdrawn 24 h after the previously administered dose of the LH-RH agonist.

*Monthly depot therapy*

All 26 patients receiving the depot formulation (3.6 mg subcutaneous/month) started treatment in the follicular phase of the menstrual cycle. Sequential endocrine studies including FSH, LH, oestradiol and progesterone were again recorded prior to treatment and at regular intervals throughout therapy (pre-treatment, days 2, 3, 5 and 7 and thereafter at weekly intervals).

To date 53 patients have been entered into the study, using daily or monthly administered Zoladex, with a minimum follow up of 6 months. The protocol of this phase one study demanded a change of therapy in the event of aggressive disease continuing to rapidly progress before the minimum time necessary to achieve ovarian suppression. Eight patients therefore have been excluded from analysis and have not been considered further, leaving 45 patients assessable for a response to therapy. Of the eight exclusions, two patients moved out of the area and so received alternative treatments, the remaining six patients were changed to non-endocrine therapies due to rapid symptomatic disease progression before completing two months on Zoladex. All these patients had hepatic or cerebral involvement and it is accepted by the authors that they may well have been unlikely to respond to endocrine manipulation.

The patients' age, disease status and sites of metastases at the presentation of advanced disease are shown in Table Ia, Ib.

*Assessment, follow-up and criteria for response*

Initial assessment included a full clinical examination with documentation of all measurable local disease and photography when appropriate. A limited skeletal survey including views of chest, skull, dorso-lumbar spine and pelvis was obtained in all patients. Isotope liver scans were performed only when clinically indicated. Haematological tests included a full blood count, ESR, urea and electrolytes, and liver function tests. The Karnofsky performance status was recorded prior to and throughout therapy.

The UICC criteria (Hayward *et al.*, 1977) demanding a 50% reduction in measurable tumour or objective radiographic evidence of regression in evaluable but non measurable disease sites (e.g. bone and lung) were employed throughout the study period to assess treatment response. The British Breast Group stipulation that any remission should be maintained for at least six months before classifying as a response was also adhered to (British Breast Group, 1974).

**Table Ia** Patients' tumour stage and sites of distant metastases. Daily subcutaneous Zoladex.

| *Patient* | *Age* | *Stage* | *Metastases* |
|---|---|---|---|
| L.G. | 46 | 4 | Lung, bone |
| D.K. | 48 | 3 | Local |
| A.H. | 45 | 4 | Bone |
| M.D. | 47 | 4 | Bone |
| G.B. | 32 | 4 | Lung |
| S.M. | 47 | 4 | Bone |
| M.M. | 43 | 3 | Local |
| P.J. | 39 | 4 | Local, bone |
| A.M. | 39 | 4 | Local, bone |
| L.S. | 40 | 3 | Local |
| S.G. | 40 | 3 | Local |
| M.B. | 45 | 4 | Lung, bone |
| C.G. | 44 | 4 | Lung, bone |
| I.E. | 47 | 4 | Local, bone |
| I.H. | 48 | 4 | Local, bone, liver |
| L.H. | 47 | 4 | Lung |
| W.G. | 47 | 3 | Local |
| J.J. | 43 | 4 | Bone |
| M.S. | 45 | 4 | Lung, bone |
| B.S. | 50 | 4 | Lung, bone |
| P.P. | 45 | 3 | Local |

**Table Ib** Patients' tumour stage and sites of distant metastases. Monthly depot formulation.

| *Patient* | *Age* | *Stage* | *Metastases* |
|---|---|---|---|
| G.B. | 46 | 4 | Lung, local |
| S.H. | 46 | 4 | Bone, liver, local |
| H.F. | 49 | 3 | Local |
| R.L. | 47 | 4 | Bone, local |
| P.C. | 50 | 4 | Lung, bone |
| J.S. | 49 | 3 | Local |
| J.K. | 46 | 4 | Lung, local |
| W.R. | 49 | 3 | Local |
| P.P. | 46 | 4 | Lung, local |
| B.S. | 38 | 4 | Lung, bone, liver |
| T.S. | 43 | 3 | Local |
| A.H. | 43 | 4 | Bone |
| M.S. | 55 | 4 | Bone, local |
| J.C. | 49 | 4 | Bone, visceral |
| J.E. | 43 | 3 | Local |
| F.C. | 44 | 4 | Bone |
| S.C. | 45 | 3 | Local |
| A.S.1 | 37 | 4 | Visceral, bone |
| J.H.1 | 45 | 4 | Bone, local |
| J.H.2 | 41 | 4 | Bone, local |
| A.W. | 29 | 4 | Bone |
| R.W. | 44 | 3 | Local |
| C.W. | 35 | 4 | Lung |
| A.S.2 | 48 | 3 | Local |

In the first 27 patients showing disease progression on Zoladex surgical oophorectomy was routinely performed in all cases fit for this procedure ($n=26$). Patients were reviewed at monthly intervals and routine haematological tests were repeated at each attendance. Skeletal surveys were repeated at three monthly intervals or more frequently if clinically indicated.

### Results

### *Endocrinological*

*Daily subcutaneous therapy* The effects of daily s.c. injections of Zoladex on circulating concentrations of LH, FSH, oestradiol and progesterone are shown in Figures 1 and 2.

Within 16 h of the administration of Zoladex there was a substantial rise in the plasma concentrations of both LH and FSH (Figure 1). On continued treatment however, basal levels of these hormones decreased and were associated with an eventual fall in circulating concentrations of both oestradiol and progesterone in all but two patients (Figure 2). With the exception of these two patients, suppression of both pituitary and ovarian hormones was maintained during active therapy with Zoladex. The levels of oestradiol and progesterone found in patients with complete ovarian suppression were equivalent to those seen in either oophorectomised or postmenopausal patients with advanced breast cancer.

In the two patients with recurrent peaks of oestradiol basal levels of cirulating LH, FSH and progesterone were maintained throughout therapy (Figure 3).

No differences in endocrine response were observed between patients receiving doses of 500 or 1000 $\mu$g daily (not illustrated).

In those patients commencing treatment in the follicular phase of the cycle ($n=12$) castrate levels of serum oestradiol and progesterone were attained

**Figure 1** Long-term effects of daily ICI 118630 on plasma LH and FSH in patients with advanced breast cancer (mean + s.e.).

**Figure 2** Long-term effects of daily ICI 118630 on plasma oestradiol and progesterone (mean + s.e.). A = postmenopausal, B = oophorectomised.

in 9 patients after one month's therapy. All 9 patients commencing treatment in the luteal phase showed some evidence of cyclical oestradiol production during the second month of treatment before castrate levels were achieved. These oestradiol peaks were, however, suppressed when compared with the mid cycle oestradiol levels found in control patients.

*Monthly depot therapy* Similar endocrinological results were obtained using the monthly depot formulation with 'castrate' levels of progesterone and oestradiol achieved in 21 of 24 assessable patients after the first month on therapy (not illustrated). The three patients refractory to the depot dosage of the LH-RH agonist used exhibited recurrent, though suppressed, peaks of oestradiol throughout a maximum of 3 months active therapy. Oestradiol concentrations were equivalent to those observed in patients starting daily subcutaneous therapy during the luteal phase of the menstrual cycle. Interestingly one of these patients with incomplete ovarian suppression had ceased to menstruate and showed objective signs of response (sclerosis in lytic bone metastases) during the first three months of therapy despite recurrent oestradiol peaks. This response was not maintained for 6 months and no response was found to subsequent oophorectomy (patient RL, Figure 2b).

### *Clinical response to Zoladex*

The ER status, clinical response to Zoladex and response to subsequent oophorectomy are shown in Table IIa, b. Fourteen of the 45 patients in whom disease was assessable on the clinical criteria used (UICC, BBG) responded partially to treatment. Three patients showed no evidence of disease

**Figure 3** Influence of daily ICI 118630 on plasma hormone levels in a patient showing recurrent peaks of oestradiol throughout therapy.

progression over 6 months therapy and in 28 patients disease progressed despite treatment.

Ten of the 14 patients showing a partial response presented with bone metastases with additional intra-abdominal involvement in two of these cases. A further patient responding to Zoladex received treatment for inoperable locally recurrent axillary nodes and the remaining three responding patients presented with stage III disease.

The oestrogen receptor status of the primary tumours was available in 38 of the 45 assessable patients. Nineteen patients were ER positive and 19 ER negative. Thirteen of 14 patients responding to Zoladex were either ER positive ($n=10$) or ER status unknown ($n=3$). The remaining eighteen patients with ER negative primary tumours all failed to respond (Table IIa, b).

Surgical oophorectomy after disease progression was performed on 26 patients. Four patients subsequently showed a response to oophorectomy having failed to respond to Zoladex. In one of these patients serum oestradiol had not been suppressed to castrate levels after 6 months on treatment (patient LH, Figure 3) and the remaining three patients had progressed from Zoladex therapy to oophorectomy after short periods of only one, two and three months respectively.

No response to oophorectomy was observed in 20 patients who failed to respond to Zoladex, in two of these patients disease remained static at 6 months. A further two patients' responses to oophorectomy after failing to respond to Zoladex were not assessable due to the addition of radiotherapy to bone metastases immediately post-operatively in one case and surgical excision of all assessable local disease in the remaining patient with stage III disease.

Two patients have undergone oophorectomy after responding to Zoladex for periods of 12 and 13 months respectively. No subsequent response was observed in either case.

The overall response rate to Zoladex in this series

**Table IIa** Oestrogen receptor status and response to daily subcutaneous treatment.

| *Patient* | *ER status* | *Response to Zoladex* ($n=21$) | *Subsequent response to Oophorectomy* ($n=18$) |
|---|---|---|---|
| L.G. | + | Nil | Nil |
| D.K. | + | Stat | — |
| A.H. | + | Nil | PR |
| M.D. | − | Nil | Nil |
| G.B. | − | Nil | Nil |
| S.M. | + | PR | Stat |
| M.M. | − | Nil | Nil |
| P.J. | − | Nil | Nil |
| A.M. | − | Nil | Stat |
| L.S. | ? | Nil | Nil |
| S.G. | ? | Nil | Nil |
| M.B. | − | Nil | Nil |
| C.G. | + | PR | Nil |
| I.E. | + | Nil | PR |
| I.H. | + | Nil | PR |
| L.H. | − | Nil | PR |
| W.G. | − | Nil | Nil |
| J.J. | + | Nil | Unassessable |
| M.S. | ? | Nil | Nil |
| B.S. | + | PR | — |
| P.P. | ? | PR | Unassessable |
| Total showing | + or unknown | 4 | 3 |
| partial response | − | 0 | 1 |

of patients, when 8 patients are excluded due to loss of follow-up or the addition of alternative treatments prior to 2 months Zoladex therapy, is therefore 31%.

### *Side effects*

Side effects relating to treatment with the LH-RH agonist were minimal and included cessation of menstruation in association with suppressed oestradiol, hot flushes (20 patients) and occasional nausea. All suppressed patients had ceased to have normal menstrual periods by two months after initiation of therapy although 7 patients experienced menstrual spotting after the second month on Zoladex.

Tumour flare, although difficult to assess in rapidly progressing advanced disease, was not observed.

### Discussion

It is apparent from this and other preliminary clinical studies that LH-RH agonists are capable of achieving responses in premenopausal patients with advanced breast cancer. The endocrinological response to Zoladex, although at present not achieved as rapidly, is quantitatively similar to that seen after surgical oophorectomy.

The initiation of daily treatment in the luteal phase of the menstrual cycle resulted in a prolongation of the time necessary to achieve complete ovarian suppression, as has been suggested in other clinical studies (Klijn, 1984). This disadvantage may have clinical implications in rapidly progressing disease.

The explanation for the inability of the LH-RH agonist to achieve complete ovarian suppression in the five patients refractory to the dosage used is not at present clear. Two patients administered their own daily subcutaneous therapy but were observed to be competent at this technique. In the remaining three patients the monthly depot formulation was administered by one author in the outpatient clinic. The failure of the LH-RH agonist did not appear to be related to either premenopausal age or body weight. In all five patients serum progesterone was suppressed to basal levels on continued treatment, an indication that anovulation had occurred.

It is of interest that one patient with incomplete ovarian suppression showed evidence of an early response to treatment in bone metastases after three months therapy, raising the possibility of a direct antitumour effect mediated by the LH-RH agonist.

**Table IIb** Oestrogen receptor status and response to monthly subcutaneous treatment.

| Patient | ER status | Response to Zoladex (n=24) | Subsequent response to Oophorectomy (n=7) |
|---|---|---|---|
| G.B. | – | Nil | Nil |
| S.H. | ? | Nil | Nil |
| H.F. | – | Nil | Nil |
| R.L. | + | Nil | Nil |
| P.C. | – | Nil | – |
| J.S. | – | Stat | – |
| J.K. | + | Nil | Nil |
| W.R. | – | Stat | – |
| P.P. | – | Nil | Nil |
| B.S. | – | Nil | – |
| T.S. | + | Nil | Nil |
| A.H. | ? | PR | – |
| M.S. | + | PR | – |
| J.C. | + | PR | – |
| J.E. | + | PR | – |
| F.C. | + | PR | – |
| J.C. | + | PR | – |
| A.S.1 | + | PR | – |
| J.H.1 | + | PR | – |
| J.H.2 | – | PR | – |
| A.W. | – | Nil | – |
| R.W. | ? | PR | – |
| C.W. | – | Nil | – |
| A.S.2 | – | Nil | – |
| Total showing | + or unknown | 9 | 0 |
| partial response | – | 1 | 0 |

Such an effect has been suggested by an *in vitro* study showing a retardation in growth of cultured mouse mammary tumour cells after the application of an LH-RH agonist (Corbin, 1982). Further evidence for a direct antitumour effect is suggested by isolated reports of responses occurring in post-menopausal women receiving treatment (Harvey *et al.*, 1981). An alternative explanation is that complete ovarian suppression may not be necessary to achieve objective remissions in all cases.

Four patients responded to oophorectomy without an apparent previous response to Zoladex. One of these patients, with lung metastases, was refractory to the dosage of the LH-RH agonist administered with serum oestradiol continuing to peak after six months therapy. This patient remained asymptomatic throughout treatment with Zoladex. The remaining three patients with bone metastases had received treatment for a maximum of only 12 weeks. The clinical responses in all patients were assessed strictly and at short intervals and so with continued therapy a response may have occurred with the use of the LH-RH agonist alone in these three patients. A retrospective review of the X-rays in two of the three patients responding to oophorectomy would suggest to the authors that a mis-classification of disease progression on Zoladex may have occurred as early signs of sclerosis were present in lytic metastases at the time of oophorectomy. Certainly their hormone profiles were comparable with oophorectomised patients.

The response rate to Zoladex of 31% is comparable to our previous experience using surgical oophorectomy and is lower than other reported series as our criteria exclude short remissions of less than six months duration.

The time course to response on Zoladex was similar to that found after surgical oophorectomy in all patients with stage III disease as was the case in those patients with bone metastases. However, in the latter situation objective responses are difficult to assess during the early stages of treatment.

The side effects relating to therapy were minimal and all patients tolerated treatment well.

Although patient numbers are small, it appears that the absence of the oestrogen receptor in the primary tumour is predictive of a failure to respond to the administration of Zoladex.

MRW is Tenovus surgical research fellow. This work was supported by a grant from the Tenovus Institute, Cardiff.

### References

BRITISH BREAST GROUP (1974). Assessment of response to treatment in advanced breast cancer. *Lancet*, **ii**, 38.

CORBIN, A. (1982). From contraception to cancer: A review of the therapeutic application of LH-RH analogues as antitumour agents. *Yale J. Brit. Med.*, **55**, 27.

HARVEY, H.A., LIPTON, A., SANTEN, R.J. & 7 others (1981). Phase II study of a gonadotrophin-releasing hormone analogue (Leuprolide) in postmenopausal advanced breast cancer patients (Abstract C-436). *Proc. Am. Assoc. Cancer Res./Am. Soc. Clin. Oncol.*, **22**, 444.

HAYWARD, J.L., CARBONE, P.P., HEUSON, J.C., KUMAOKA, S., SEGALOFF, A. & RUBENS, R.D. (1977). Assessment of response to therapy in advanced breast cancer. A project of the programme of clinical oncology of the International Union Against Cancer, Geneva, Switzerland. *Cancer*, **39**, 1289.

KENNEDY, B.J., MIELKE, P.W., FORTUNY, I.E. (1964). Therapeutic castration versus prophylactic castration in breast cancer. *Surg. Gynecol. Obstet.*, **118**, 524.

KLIJN, J.G.M. (1984). Long-term LHRH-agonist treatment in metastatic breast cancer as a single treatment and in combination with other additive endocrine treatments. *Med. Oncol. & Tumor. Pharmacother.*, **1**, 123.

MAYNARD, P.V. & NICHOLSON, R.I. (1979). Effects of high doses of a series of new luteinising hormone-releasing hormone analogues in intact female rats. *Br. J. Cancer*, **39**, 274.

NICHOLSON, R.I. & MAYNARD, P.V. (1979). Anti-tumour activity of ICI 118630, a new potent luteinising hormone-releasing hormone agonist. *Br. J. Cancer*, **39**, 268.